# Bibliometric analysis of global research output on antimicrobial resistance in the environment (2000–2019)

**DOI:** 10.1186/s41256-020-00165-0

**Published:** 2020-08-03

**Authors:** Waleed M. Sweileh, Ahmad Moh’d Mansour

**Affiliations:** 1 Department of Physiology, Pharmacology/Toxicology, College of Medicine and Health Sciences, An-Najah National University, Nablus, Palestine; 2 Business Faculty, Al-Balqa Applied University, Amman, Jordan

**Keywords:** Antimicrobial resistance, Environment, Research activity, One health

## Abstract

**Background:**

Antimicrobial resistance (AMR) is a global health threat that requires a “One Health” approach. Of the One Health triad, the environmental component is the most dynamic and most neglected. Therefore, the objective of the current study was to assess and analyze global research activity on AMR in the environment.

**Methods:**

This was a bibliometric descriptive study of publications on AMR in the environment. Publications were retrieved using SciVerse Scopus for the study period from 2000 to 2019. The search query was developed using terms and phrases related to the topic. The retrieved publications were analyzed for specific bibliometric indicators including annual growth, citation analysis, key players, research output for each world regions, research themes, and occurrences of different drug classes of antimicrobials. Visualization maps including research collaboration were created using VOSviewer program. The Hirsch (*h*) index was used to assess scientific impact.

**Results:**

There were 2611 research articles based on the implemented research query. The retrieved documents had an average of 22 citations per document and an *h*-index of 122. The annual number of publications showed a steep increase from 2011 to 2019. The major research themes in the field were (1) dissemination and abundance of antibiotic-resistant genes and (2) detection of bacterial strains or antibiotic residues in various environmental isolates. The bulk of the retrieved articles (*n* = 899; 34.4%) originated from the European region. China led with 598 (22.9%) documents. Four of the top 10 active institutions were in China. The top 10 active countries had relatively inadequate international research collaboration. The most commonly encountered antibiotic drug classes in the retrieved articles were penicillin/cephalosporin (*n* = 1152 occurrences). The most frequently encountered pathogen in the retrieved publications was *E. coli* (*n* = 666). The *Science of the Total Environment* journal was the most prolific journal with 139 (5.3%) publications.

**Conclusion:**

Scientific literature on the AMR in the environment has witnessed a steep growth lately with a leading role of China and Chinese institutions. Data on AMR in the environment need to be collected from all world regions including the Eastern Mediterranean and African regions through research collaboration and funding of research in this field.

## Background

Antimicrobial resistance (AMR) is a global health threat [1] that requires a “One Health” approach [2–4]. The “One Health” approach recognizes that the health of people is closely connected to the health of animals and the environment [5]. The “One Health” approach has been adopted by the United Nation (UN) in the fight against AMR and in the roadmap to achieve the third goal in Sustainable Development Goals 2030 (SDGs) about health and well-being [6, 7]. Therefore, a holistic approach is required to confront global public health problems, such as AMR, that is present in humans, animals, and the environment [8, 9]. Of the One Health triad, the environment component is the most dynamic and most neglected [10].

The AMR is the consequence of multiple factors related to inappropriate and overuse of antimicrobial agents in humans, animals, food chain, and the exposure of the environment to antimicrobial agents [11]. Antimicrobials end up in the environment through industrial, pharmaceutical, and hospital effluents as well as human and animal wastes [12]. For example, antimicrobial agents are found in large quantities in aquatic environments, sewage and wastewater treatment plants [12, 13]. There are several routes for transmission of antibiotic-resistant bacteria from humans and animals to the environment, and since all environmental compartments are connected, these resistant bacteria can be transmitted back to humans through water, animal-producing food, vegetables, fruits, and other edible agricultural products [14–16].

Assessment of research activity on AMR in the environment is used to achieve several goals: (1) evaluate the progress and growth of literature dedicated to discuss AMR in a “One Health” approach; (2) help draw a global map on regions with a poor contribution to the One Health approach in combating AMR and therefore be subject to encouragement and collaboration from various international organizations and academic/research institutions which strengthens the collaborative and multi-sectoral approach recommended by the “One Health”; (3) help identify prominent research/academic institutions involved in this field and direct international funding to such institutions, (4) help developing strategic national decisions concerning national action plans in combating AMR; (5) help microbiologists, pharmacologists, and clinicians understand what pathogens and antimicrobials that are being cycled at the human-animal-environment interface; and (6) establish the role of various scientific subject areas in the published literature on AMR and the environment.

Research activity is usually evaluated and assessed using bibliometric analysis, which is defined as the quantitative analysis of science using statistical methods and mapping techniques. Bibliometric analysis is different from systematic reviews and scoping reviews. In a bibliometric analysis, documents are retrieved using a single database and analyzed quantitatively and qualitatively for specific bibliometric indicators [17, 18]. Bibliometric analysis is not inclusive of grey literature. In systematic reviews, a research question is answered using a limited number of publications selected from different databases, including grey literature. In systematic reviews, but not in bibliometric analysis, the initially retrieved documents include a large percentage of duplicate documents that are filtered using certain selection methods. In systematic reviews, researchers might follow up with the analysis and carry out meta-analysis, which is not the case in the bibliometric analysis [19, 20]. For scoping reviews, it is defined as a preliminary assessment of potential size and scope of available research literature, usually including ongoing research, and aims to identify nature and extent of research evidence [21, 22]. Bibliometric studies on various aspects of AMR such as carbapenem resistance, antimalarial drug resistance, anti-tuberculosis drug resistance, uropathogen resistance, and several others have been published [23–27]. However, no bibliometric studies have been published on AMR in the environment. Therefore, the current study was undertaken to assess and analyze the global research activity on AMR in the environment.

## Methods

### Database selection

In the current study, Scopus database was used as a tool to retrieve the relevant documents. Several advantages of Scopus make it suitable for such studies. First, Scopus provides basic and advanced search options and allows for the use of different Boolean operators, which allow for the development of comprehensive search query. Second, Scopus has many analytic functions such as citation and subject analysis. Third, quantitative analysis regarding active key players can be extracted from Scopus directly. Fourth, Scopus allows simple export of Scopus data to Microsoft Excel or any other program such as VOSviewer for further analysis and mapping. Fifth, Scopus is superior to Pubmed because Pubmed is 100% inclusive in Scopus. Sixth, Scopus is larger and has more non-English scientific journals than Web of Science [28]. This is important because in the current study no language restriction was imposed. All documents published in Scopus must have an English title and abstract, which enables researchers to confirm the content of non-English documents through title/abstract content.

### Search strategy

The key step in bibliometric studies is the development of a comprehensive search query with a high tested validity to ensure accurate analysis and outcome. The search query in the current study was developed after reviewing both scientific publications and grey literature on the topic to develop a bank of relevant keywords to be used in the search query [29–31]. In the current study, the search query was based on two search scenarios joined by the Boolean operator “AND”. The first scenario was dedicated to retrieve documents on AMR: “antibiotic resistan*” or “antimicrobial resistan*” or “antibacterial resistan*” while the second scenario was dedicated for documents related to the environment (e.g. water or soil or waste or sludge or aquatic or manure or sewage or effluent or “wild animal*” or “f*ecal contamination” or vegetable* or fruit* or river). The asterisk and quotation marks were used in the research query to increase comprehensiveness and accuracy. Both scenarios were then combined. The flow diagram of study selection is shown in Supplementary material 1 while the keywords used in the search strategy are shown in Supplementary material 2. The PRISMA checklist is shown in Supplementary material 3.

### Refining the retrieved documents

The retrieved documents were limited to journal research articles only. Therefore, books, notes, letters, editorials, and errata were excluded. The purpose of this step was to focus on original research rather than scientific publications in the form of commentaries, notes, and others. The study period was limited from 2000 to 2019. The choice of the study period was based on the understanding that AMR became internationally a serious health problem in the past two decades. Furthermore, the initial assessment of the literature showed that there were few publications on AMR from the perspective of the environment before the year 2000. No language restriction was imposed on the retrieved literature.

### Validation of the search strategy

In the current study, the development of the search query was continuously fine-tuned until the top 200 cited documents in the retrieved literature were free of any false-positive results. Furthermore, the search query was tested for missing data (false-negative) by adopting a previously published method which relies on the correlation between the retrieved research output for active authors and their actual research output in this field [27].

### Data export and analysis

Retrieved data were exported from Scopus to Microsoft Excel for analysis and table presentation. Graphics were created using Statistical Package for Social Sciences (SPSS, version 21). Geographical distribution of publications was carried out using the WHO geographical classification: the region of the Americas; the European region, the Eastern Mediterranean region, the African region, the South-Eastern Asia region, and Western Pacific region). Visualization maps were created using VOSviewer program [32]. International research collaboration among active countries was assessed using the “link strength” indicator extracted from visualization maps. The link strength is a measure of the strength of research collaboration between any two countries. The link strength is proportional to the thickness of connecting lines between countries. The higher the value of link strength, the thickness of the connecting line, the stronger the research collaboration [33].

The scientific impact of publications from different countries can be compared using the normalized citation value of the published documents from each country. The normalized citation value was obtained from the network visualization maps created by VOSviewer. The normalized citation of the research output of any specific country is proportional to the node size for that country in the normalized citation visualization map. The larger the node size of the country, the higher the scientific map of its research output [33]. The Hirsch-index (*h*-index) was used to measure the scientific impact of authors, institutions, countries, and a body of literature. The *h*-index is defined as the maximum value of h such that the given author/journal has published h papers that have each been cited at least h times [34]. The *h*-index is proportional to research productivity and the number of citations.

### Bibliometric indicators

In the current study, the following bibliometric indicators were presented: (1) volume and growth of publications on AMR in the environment over the past two decades; (2) research output from different world regions; (3) subject areas of literature on AMR in the environment; (4) most active countries, institutions, journals, and funding agencies involved in publishing scientific articles on AMR in the environment; and (5) the antimicrobial drug classes and pathogens mostly encountered in the retrieved literature.

## Results

### Characteristics of the retrieved publications

The search query implemented in Scopus database retrieved 2611 research articles. The majority of the retrieved articles (*n* = 2425; 92.9%) were research articles, review articles (*n* = 164; 6.3%), and conference papers (*n* = 22; 0.8%). The language in the majority of the retrieved articles was English (*n* = 2470; 94.6%) followed by Chinese (*n* = 80; 3.1%) and Spanish (*n* = 10; 0.4%). Other less commonly encountered languages included Portuguese, German, French, Korean, and Russian. Less than one-third of the retrieved research articles (*n* = 849; 32.5%) were available as open access for readers.

### Subject areas of the retrieved documents

The retrieved articles belonged to the following subject areas: environmental sciences (*n* = 1234; 47.3%), immunology/microbiology (*n* = 799; 30.6%), medicine (*n* = 652; 25.0%), agricultural and biological sciences (*n* = 555; 21.3%), biochemistry/genetics/molecular biology (*n* = 486; 18.6%), and pharmacology/toxicology (*n* = 231; 8.8%).

### Annual growth of publications

**Fig. 1:**
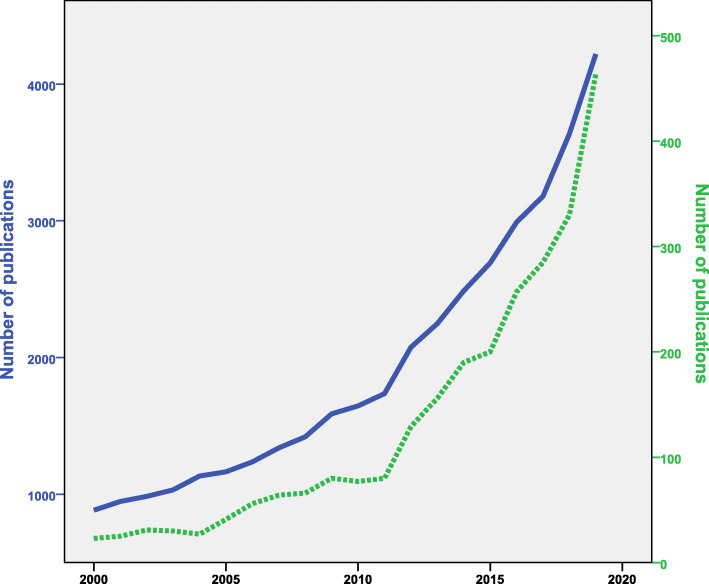
Annual growth of publications on antimicrobial resistance in the environment. The dashed green line represents the annual growth of AMR in the environment while the blue line represents the annual growth of publications on AMR in general

The graph for annual growth of publications showed two phases: a slow phase from 2000 to 2010 and a steep phase from 2011 to 2019. Figure 1 is a representation of the annual growth of publications where the blue line represented the growth of worldwide publications on AMR while the green dashed line represented the annual growth of publications on AMR in the environment. The graph was created on a dual Y-axis to compare the two lines. The figure showed that the gap between the two lines is narrowing with time indicative of greater worldwide interest in the role of environment in the propagation of AMR. In the year 2000, publications on AMR in the environment constituted 2.6% of the overall publications on AMR. However, in 2019, publications on AMR in the environment constituted 11.0% of the overall publications on AMR. The search query on AMR alone found 38,639 publications and therefore the research on the AMR in the environment constituted 6.7% (*n* = 2611) of the overall AMR literature.

### Research themes

**Fig. 2:**
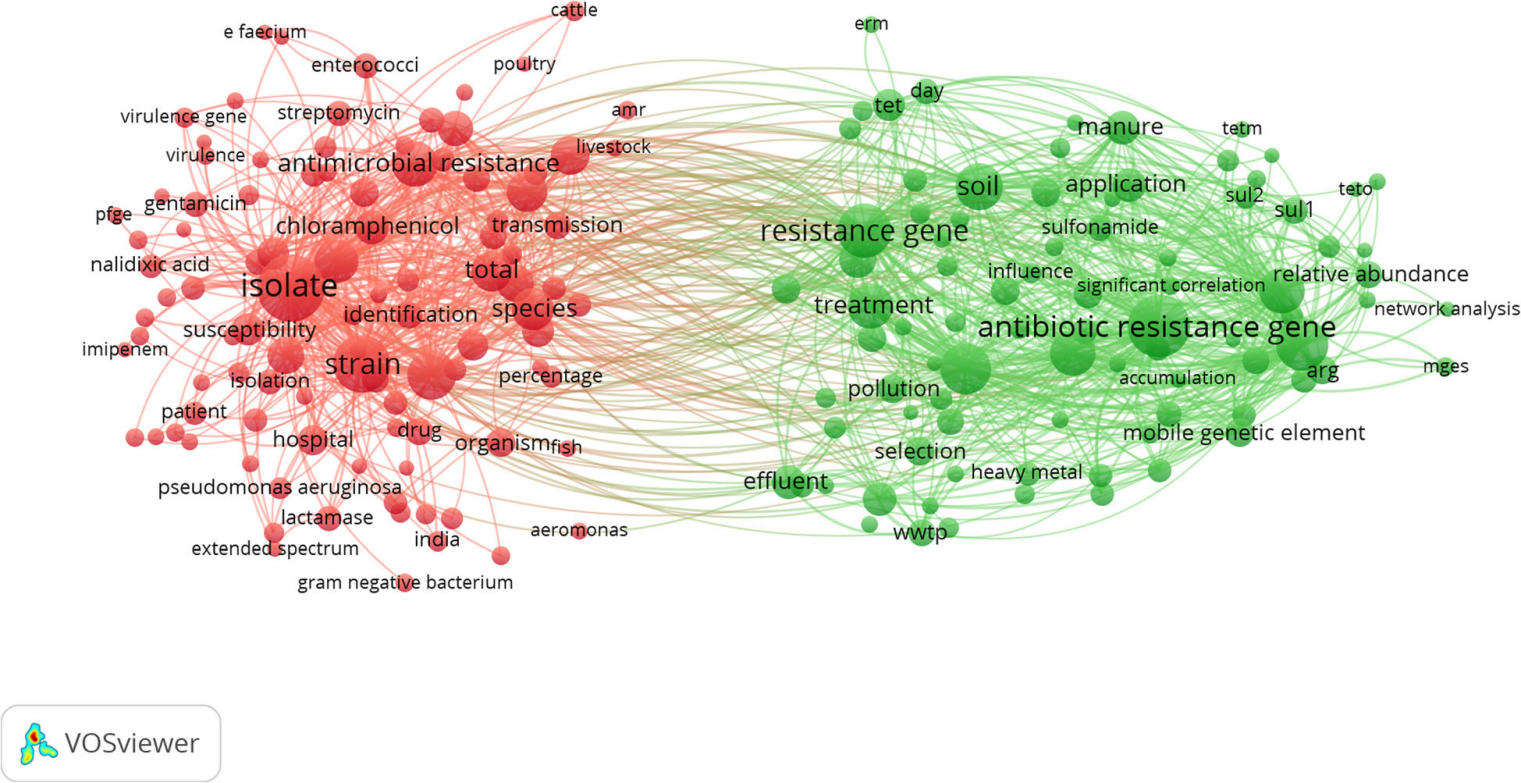
Network visualization map of most frequent terms in titles/abstracts of the retrieved literature on AMR in the environment (2000–2019). The two clusters represent the major research themes in the retrieved literature

Visualization of terms in the titles/abstracts and having minimum occurrences of 50 times showed that there were two major research themes in the field (Fig. 2). The first theme (green) represented research on the dissemination and abundance of antibiotic-resistant genes in the environment while the second theme (red) represented research on detection of bacterial strains or antibiotics in environmental isolates.

### Antimicrobials and pathogens

Analysis of indexed keyword occurrences showed that the most commonly encountered antibiotic drug classes were penicillin/cephalosporin (*n* = 1152 occurrences) followed by aminoglycosides (*n* = 1058) (Table 1). Analysis also showed that the most commonly encountered pathogens were *E. coli* (*n* = 666), *Pseudomonas aeruginosa* (*n* = 172), *Staphylococcus aureus* (*n* = 154), *Enterococcus faecalis/faecium* (*n* = 134), and *Salmonella* (*n* = 119).

**Table 1:** Top ten frequent drug classes encountered in the retrieved documents (AMR in the environment) during the study period from 2000 to 2019

Rank	Drug class	Occurrences (%) ***N*** = 2611
1	*Penicillin and Cephalosporines (B-Lactams)*	1152 (44.1)
2	*Aminoglycosides*	1052 (40.3)
3	*Tetracycline*	958 (36.7)
4	*Quinolones/ Fluoroquinolones*	852 (32.6)
5	*Folate antagonists*	831 (31.8)
6	*Macrolids*	462 (17.7)
7	*Chloramphenicol*	409 (15.7)
8	*Carbapenems (B-Lactams)*	227 (8.7)
9	*Glycopeptides (vancomycin,)*	222 (8.5)
10	*Polypeptide (Colistin/Polymyxin)*	42 (1.6)

*AMR* antimicrobial resistance

### Geographic distribution of the retrieved documents

Authors from the European region participated in 899 (34.4%) of the retrieved literature. Authors from the region of the Americas participated in 756 (29.0%) articles while authors from the Western Pacific region participated in 743 (28.5%) articles. The least contribution was made by authors from the South-Eastern Asian region (*n* = 259; 9.9%), the Eastern Mediterranean region (*n* = 179; 6.9%) and the African region (*n* = 151; 5.8%).

### Active countries

**Fig. 3:**
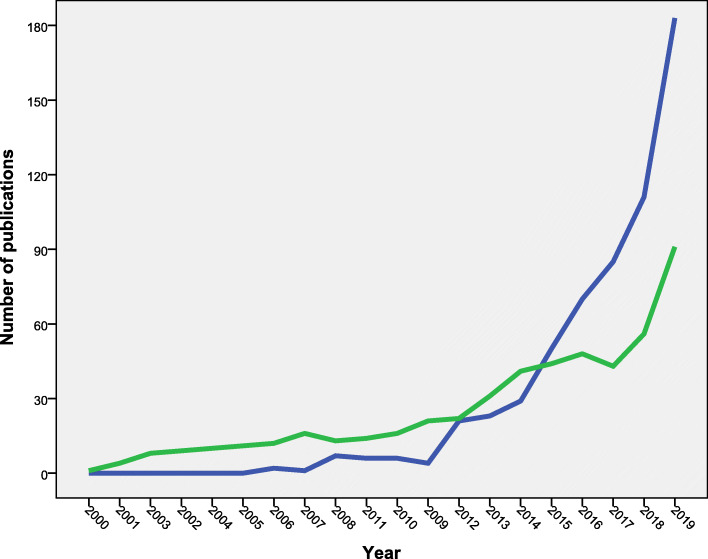
Comparison of the annual growth of publications on antimicrobial resistance in the environment. The green line represents the annual growth of publications from the USA while blue line represents the annual growth of publications from China

Table 2 showed the top 10 active countries. China led with 598 (22.9%) articles followed by the USA (*n* = 511; 19.6%). The top 10 active countries contributed to 2082 articles (79.7%). The top active countries included one in South America, two in Northern America, five in Europe, one in the Western Pacific region, and one in South-Eastern Asian region. When data were normalized by income and population size, India ranked first followed by China. Figure 3 compared the growth of publications from the USA and China. Publications from China started later than that from the USA. However, the productivity from China exceeded that from the USA in the last 4 years.

**Table 2:** Top ten active countries in AMR in the environment research (2000–2019)

Rank	Country	Frequency (%) ***N*** = 2611	Number of publications/ GDP (nominal) per capita (10^**− 3**^)*
1	China	598 (22.9)	59.2
2	United States	511 (19.6)	7.8
3	India	196 (7.5)	89.1
4	United Kingdom	147 (5.6)	3.6
5	Canada	132 (5.1)	2.9
6	Germany	129 (4.9)	2.8
7	Spain	110 (4.2)	3.7
8	Brazil	101 (3.9)	11.5
9	Portugal	90 (3.4)	3.9
10	France	68 (2.6)	1.6

(*) Number of publications/GDP (nominal) per capita was calculated by dividing the number of publications by the GDP (nominal) per capita for each country. Data on GDP (nominal) per capita was obtained from the World Bank data

*AMR* antimicrobial resistance

### International research collaboration

**Fig. 4:**
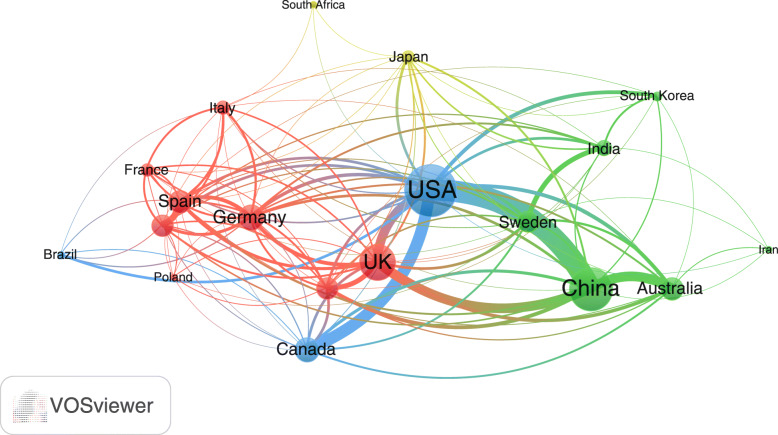
Network visualization map of international research collaboration among countries with minimum research output of 50 documents on AMR in the environment. The thickness of the connecting lines represents the strength of research collaboration between any two countries. The connecting line between the USA and China represents the strongest research collaboration due to it thickness relative to other lines

Countries with a minimum contribution of 50 documents were visualized to assess international research collaboration among active countries (Fig. 4). For research collaboration, the strongest was between China and the USA (link strength = 58) followed by that between the USA and Canada (link strength = 38). The link strength was less than 10 for most countries indicative of inadequate international research collaboration.

### Citation analysis

**Fig. 5:**
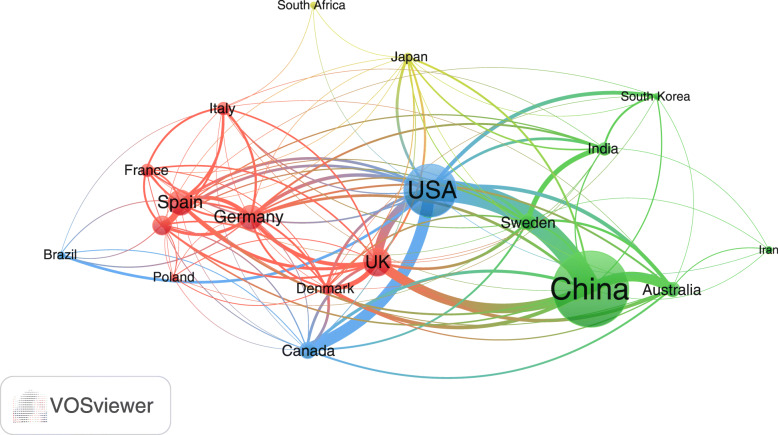
Network visualization map of scientific impact of publications from countries with minimum research output of 50 documents. Publications from China have the highest scientific impact due to the size of the node representing China

The retrieved documents received 75,331 citations, an average of 22 citations per document and an *h*-index of 122. Publications from China had the highest scientific impact followed by those from the USA as measured by normalized citations (Fig. 5).

### Active institutions

The leading role of China in this field was reflected in the findings that four of the top 10 academic institutions/organizations were based in China. The top active institution was the *Chinese Academy of Sciences* (*n* = 177; 6.8%) followed by *Agriculture et Agroalimentaire Canada* (*n* = 52; 3.2%), and *Ministry of Education China* (*n* = 44, 1.7%). Other active institutions were listed in Table 3.

**Table 3:** List of top ten active institutions in AMR in the environment (2000–2019)

Rank^**a**^	institution	Frequency (%) ***N*** = 2611	Country
1	*Chinese Academy of Sciences*	177 (6.8)	China
2	*Agriculture et Agroalimentaire Canada*	52 (2.0)	Canada
3	*Ministry of Education China*	44 (1.7)	China
3	*Universidade Catolica Portuguesa, Porto*	44 (1.7)	Portugal
5	*USDA Agricultural Research Service, Washington DC*	41 (1.6)	USA
6	*Northwest A&F University*	40 (1.5)	China
7	*Tsinghua University*	38 (1.5)	China
8	*Universidade do Porto*	32 (1.2)	Portugal
9	*Michigan State University*	29 (1.1)	USA
10	*Zhejiang University*	27 (1.0)	China
10	*The University of Hong Kong*	27 (1.0)	Hong Kong

*AMR* antimicrobial resistance

^a^Two equal institutions were given the same rank and one place in the ranking order is skipped

### Active journals

The top 10 active journals are listed in Table 4. The active journals published 689 (26.4%). *Science of The Total Environment* journal was the most prolific journal with 139 publications followed by *Frontiers in Microbiology* with 89 publications. The top 10 active journals included six journals in the field of environmental sciences, three in microbiology, one in environmental microbiology, and one miscellaneous journal. The *Science of The Total Environment* journal received the highest normalized citation per document. All journals in the top 10 list ranked by Scopus as Q1 and all of them were affiliated with countries in the top 10 list, mainly with the USA, the UK, and the Netherlands.

**Table 4:** Top ten active journals in AMR in the environment research (2000–2019)

Rank^**a**^	Country	Frequency (%) ***N*** = 2611	Rank	Affiliation
1	*Science of The Total Environment*	139 (5.3)	Q1	Netherlands
2	*Frontiers in Microbiology*	82 (3.1)	Q1	Switzerland
3	*Applied and Environmental Microbiology*	73 (2.8)	Q1	USA
4	*Environmental Pollution*	69 (2.6)	Q1	UK
5	*Water Research*	64 (2.5)	Q1	Netherlands
6	*Environmental Science and Technology*	60 (2.3)	Q1	USA
7	*Environmental Science and Pollution Research*	55 (2.1)	Q1	Germany
7	*Plos One*	55 (2.1)	Q1	USA
9	*Environment International*	48 (1.8)	Q1	UK
10	*Journal of Applied Microbiology*	44 (1.7)	Q1	UK

Q1: first quartile (highest rank)

^a^Two equal journals were given the same rank and one place in the ranking order is skipped

### Funding agencies

Of the retrieved articles, 1973 (75.5%) publications were funded projects. The National Natural Science Foundation of China (315; 12.1%) was the most active funding agency in this field.

## Discussion

The current study aimed to assess the scientific literature on AMR in the environment. The contamination of the environment with antibiotic-resistant bacteria, antibiotic-resistant genes, and residues of antibiotics constitute a global public health challenge due to their associated risk to human health [35]. Both the WHO and European action plans to tackle AMR mentioned the environment and called for developing guidelines and standards to minimize environmental pollution with antibiotics from various facilities [36, 37]. The presence of resistant bacteria and bacterial resistant genes in the environment has an active role in the propagation of AMR [38].

The current study showed that literature on AMR in the environment is growing. The growth of the publications was enhanced by the increasing recognition of scientists and international organizations that protecting the environment is the responsibility of a wide and diverse group of stakeholders [39]. The current study clearly showed that the retrieved documents belonged to a wide range of subject areas including environment, pharmacology, microbiology, and medicine suggesting that authors from different scientific backgrounds being involved in publishing the retrieved documents. A bibliometric study on pharmaceuticals in the environment showed exponential growth of literature in this field which spans multidisciplinary subjects [40].

The current study indicated that China and the USA were leaders in this field. China is one of the world regions where gram-negative bacterial resistance is severe and threatening human and animal health [41]. The irrational use of antimicrobials and the unregulated use of antimicrobials in food-producing animals in China are the main driving factors for the AMR crisis in China [42, 43]. A recently published research paper indicated that India and China are hot spots of antimicrobial resistance in animals and that Brazil and Kenya are newly emerging hot spots [44]. In the current study, both China and India ranked first and third respectively in the number of publications concerning AMR in the environment. Authors from the United States, the UK, Canada, and Germany ranked second, fourth, fifth, and sixth respectively. This result is not surprising since these countries are leading the world in research, including medicine [23, 24, 45]. The current study showed that Brazil ranked 9th in research activity on AMR in the environment. This might be due to the overuse of antimicrobials in animal-producing food. A study expected an increase in global consumption of antimicrobials by 67% by 2030 and that consumption in Asia is projected to increase to 51,851 tons which represents 82% of global antimicrobial consumption in food animals in 2010 [46]. The authors of the paper reported that in 2010 China (23%), the United States (13%), Brazil (9%), India (3%), and Germany (3%) had the largest share of global use while in 2030 China (30%), the United States (10%), Brazil (8%), India (4%), and Mexico (2%) will have the largest share of antimicrobial use in animal food [46].

The current study indicated the presence of two major themes in the retrieved literature: antibiotic in environmental isolates and abundance of resistant bacterial genes in various environmental compartments. The current study indicated that at least nine different antibiotic drug classes were encountered in the retrieved literature. These antibiotics have been reported in various environmental compartments in various parts of the world [47–49]. The massive non-clinical use of antibiotics led to the presence of these antibiotics in large quantities in human-made environments such as sewage and wastewater treatment plants and marine environments [12, 13]. The presence of antibiotic residues in the environment triggers bacterial mutation favoring resistance due to selective pressure. Antibiotic-resistant genes are disseminated in the environment through a horizontal or vertical transfer with the potential of exchange between environmental and pathogenic bacteria [50]. Humans become exposed to these resistant pathogens by direct or indirect contact with the environment or animals.

In the current study, environment journals and water research journals were in the top 10 active list. A study showed that *Chemosphere* journal published the largest number of articles followed by the *Science of the Total Environment* journal while *Water Research* journal ranked the first in the category list of water resources and the second in the category of environmental / engineering [51]. In the current study, none of the journals in the top active list was in public health or general medicine. The majority of retrieved documents was in the subject area of environmental sciences followed by microbiology/immunology. The link between environment and human health is well established and the development of AMR in the environmental components should be considered a national and global public health priority [52].

The current study showed relatively high citations and *h*-index indicative of a large number of readers interested in the subject. The *h*-index of documents on AMR in the environment was higher than that reported for literature on strongyloidiasis [53], epidermal parasitic skin diseases [54], and AIDS-related stigma [54] but equal to that on carbapenem resistance [23]. The high *h*-index value even in the presence of limited international research collaboration is evidence of the importance of the topic itself rather than self-citations. The high number of citations was also enhanced by the involvement of highly influential journals in publishing documents in this field.

The current study is the first bibliometric study to study this topic and present detailed information on research trend and growth on this emerging topic. However, the fact that we used Scopus to retrieve relevant literature with the exclusion of grey literature and publications in un-indexed journals make the data uncomprehensive. The term environment is very broad. In the search strategy, we used the maximum known terms but not all possible ones. Third, the validation of the study was based on previously used validation method. However, a small percentage of error remains a possibility and might be reflected on the listing of active players in the field. This small percentage of error is due to the fact that not all retrieved studies can be assessed and confirmed for its relevance to the topic of the study.

## Conclusion

Antimicrobial resistance is a global and multidimensional problem that needs a comprehensive, global, and “One Health” approach. In the current study, scientific literature on the AMR in the environment was assessed and analyzed. Literature in this field has witnessed a rapid and steep growth in the last decade. China and Chinese institutions played a leading role in this field. Two major themes dominated the literature on AMR and the environment: the presence of antibiotics in various environmental compartments and the abundance and dissemination of antibiotic-resistant genes in the environment. The literature in this field was mainly within environmental and microbiology subject areas. Information regarding AMR and the environment need to be obtained from all world regions including the Eastern Mediterranean and African regions. Pathogenic bacteria and resistant genes frequently encountered in the retrieved literature need to be considered in national and global policies and environmental technologies implemented.

## Supplementary information

**Additional file 1.**

**Additional file 2.**

**Additional file 3.**

## Data Availability

All data presented in this manuscript are available on Scopus database using the search query listed in the methodology section.

## Abbreviations

AMR: Antimicrobial resistance
WHO: World Health Organization
USA: United States of America
UK: United Kingdom
