# Supplementary material for: Bibliometric analysis of global research output on antimicrobial resistance in the environment (2000–2019)
Source: Glob Health Res Policy. 2020 Aug 3;5:37. doi: 10.1186/s41256-020-00165-0 (PMC7398083; doi:10.1186/s41256-020-00165-0)
Supplement: Supplementary file 1 — Additional file 1. [file 41256_2020_165_MOESM1_ESM.docx]

**Supplementary material 1**

**Bibliometric analysis of global research output on antimicrobial resistance in the environment (2000 - 2019)**

Flow diagram of study selection using Scopus database

Number of documents on environmental aspects

**N=2385864**

Number of documents on antimicrobial resistance/antibiotic resistance

**N=56989**

**N=3282**

**Limit to journal research articles (N=3088)**

**Limit duration from 2000 to 2019 (N=2611)**

**Exclude 116 documents on plants or drug discovery**

Number of documents on antimicrobial resistance/antibiotic resistance and environmental aspects combined

**N=3398**
