# Supplementary material for: Bibliometric analysis of global research output on antimicrobial resistance in the environment (2000–2019)
Source: Glob Health Res Policy. 2020 Aug 3;5:37. doi: 10.1186/s41256-020-00165-0 (PMC7398083; doi:10.1186/s41256-020-00165-0)
Supplement: Supplementary file 2 — Additional file 2. [file 41256_2020_165_MOESM2_ESM.docx]

**Supplementary material 2**

**Bibliometric analysis of global research output on antimicrobial resistance with the perspective of the environmental health (2000 - 2019)**

Database: SciVerse Scopus

Keywords and phrases used in the search strategy

| **Number of documents retrieved** | **Keywords used** | **Step** |
| --- | --- | --- |
| 56989 | title-abs (antibiotic or antimicrob* or antibacterial) and title (resistan*) and title-abs-key ("antibiotic resistan*" or "antibacterial resistance" or "antimicrobial resistan*" or "*drug resistan*") | #1 |
| 2,385,864 | title ( "environment*" or "soil" or "water" or "air pollut*" or "wild animals" or "animal feeds" or feedlots or "manures" or "aquaculture" or "waste water*" or "sewage" or "animal hospitals" or "urban areas" or "hospital effluent*" or wildlife or river or waste or vegetables or fruits or plants or "fecal contamination" or "fecal waste" or watershed or groundwater or "treatment plant" ) and all ( environment* or water or soli )) and abs ( experiment* ) | #2 |
| 3398 | Combine (#1 AND #2) | #3 |
| 3282 | exclude title-abs-key ("therapeutics" or phytochemical* or "plant extract" or "drug discovery" or "aids" or "hiv" or "influenza") | #4 |
| 3088 | Limit to journal research articles | #5 |
| 2611 | Limit to documents from 2000 to 2019 | #6 |
